# Isolation of Uncultured Bacteria from Antarctica Using Long Incubation Periods and Low Nutritional Media

**DOI:** 10.3389/fmicb.2017.01346

**Published:** 2017-07-14

**Authors:** Andre A. Pulschen, Amanda G. Bendia, Ashwana D. Fricker, Vivian H. Pellizari, Douglas Galante, Fabio Rodrigues

**Affiliations:** ^1^Instituto de Química, Universidade de São Paulo Butantã, Brazil; ^2^Departamento de Oceanografia Biológica, Instituto Oceanográfico, Universidade de São Paulo Butantã, Brazil; ^3^Laboratório Nacional de Luz Síncrotron, Centro Nacional de Pesquisa em Energia e Materiais Campinas, Brazil; ^4^Departamento de Química Fundamental, Instituto de Química, Universidade de São Paulo Butantã, Brazil

**Keywords:** uncultured, Antarctica, *Solirubrobacterales*, *Thermoleophilia*, slow-growing bacteria

## Abstract

Uncultured microorganisms comprise most of the microbial diversity existing on our planet. Despite advances in environmental sequencing and single-cell genomics, in-depth studies about bacterial metabolism and screening of novel bioproducts can only be assessed by culturing microbes in the laboratory. Here we report uncultured, or recalcitrant, microorganisms from an Antarctic soil sample, using relatively simple methods: oligotrophic media, extended incubation periods, observation under stereo microscopy, and selection of slow-growing bacteria. We managed to isolate several rare microorganisms belonging to infrequently isolated or recently described genera, for example *Lapillicoccus, Flavitalea, Quadrisphaera, Motilibacter*, and *Polymorphobacter.* Additionally, we obtained isolates presenting 16S rRNA sequence similarity ranging from 92.08 to 94.46% with any other known cultured species, including two distinct isolates from the class *Thermoleophilia*, that although common in Antarctic soils (as identified by metagenomics), was never reported to be isolated from such samples. Our data indicates that simple methods are still useful for cultivating recalcitrant microorganisms, even when dealing with samples from extreme environments.

## Introduction

Most environmental microorganisms are still classified as uncultivable (Hug et al., 2016), or using the best definition, “yet-to-be cultivable,” considering that all organisms should be able to grow and divide, and therefore, capable of growing under the proper conditions (Lewis et al., 2010; Vartoukian et al., 2010; Stewart, 2012).

Reasons for failing to grow most of the existing microorganisms in the laboratory vary. Some are simple, for example failing to supplement the correct nutrients (Kopke et al., 2005), using the wrong pH, insufficient incubation periods (Janssen et al., 2002; Davis et al., 2011), or the use of extremely rich-media (which favors fast growing cells, that can overgrow slower microorganisms). Some are more complex, such as the need for specific growth signals (Bruns et al., 2002; Nichols et al., 2008), dependence on other microorganisms (D’Onofrio et al., 2010; He et al., 2015), or development as microcolonies (Davis et al., 2011).

Many reasons justify the interest in growing “uncultivable” organisms. Features such as determination of growth preferences, consumption or production of environmental metabolites (involved in nutrient cycling), physiological characteristics (cell size, morphology, pigmentation, motility, etc), and virulence profiles can only be properly studied by growing cells under laboratory conditions (Wade, 2002; Vartoukian et al., 2010; Stewart, 2012; Baker et al., 2015). Furthermore, growth of recalcitrant microbes has potential for biotechnological applications (Newman, 2016), for example the recently discovered bacterial species capable of degrading the environmental pollutant Polyethylene terephthalate (Yoshida et al., 2016) or the discovery of a novel antibiotic class, teixobactin, produced by a bacterium from a previously undescribed genus (Ling et al., 2015). Finally, features like resistance to environmental challenges, such as radiation, desiccation, extremely low or high temperatures also requires microbial isolation to be proper studied. Such investigations are important not only to improve our knowledge toward microbial physiology, but also to improve our knowledge about the limits of life, a topic that is of great interest for Astrobiology (Rothschild and Mancinelli, 2001; Mykytczuk et al., 2013; Pulschen et al., 2015; Paulino-Lima et al., 2016).

In order to assess and grow recalcitrant microorganisms, several technologies and methods have been developed. One of the most well-known techniques relies in using diffusion chambers (Kaeberlein, 2002; Ferrari et al., 2005), which allow the passage of nutrients and metabolites from the environment to the trapped microorganisms inside the chamber, achieving special requirements for the growth of certain cells. The iCHIP method (isolating chip) is a similar technique that optimizes the principles of diffusion chambers and it has already been used to isolate several novel bacteria (Nichols et al., 2010; Ling et al., 2015). Whole-genome sequencing can be used to develop isolation strategies based on the metabolic information existing in the genomic DNA (Tyson et al., 2005; Gtari et al., 2015; Wurch et al., 2016). Metatranscriptomic analysis has also been used to direct culturing of recalcitrant bacteria (Bomar et al., 2011). However, many of such techniques are time-consuming and/or imply a high cost. Some other techniques for retrieving hard-to-culture microbes are much simpler, such as the choice of a different solidifying agent (Tamaki et al., 2009), longer incubation periods, growth in low nutrient media (Janssen et al., 2002; Sait et al., 2002; Davis et al., 2005), and separate preparation of media components (Tanaka et al., 2014). Most of the earth’s biosphere (over 80%) remains permanently cold, with temperatures below 5°C (Rodrigues and Tiedje, 2008; Cavicchioli et al., 2011; De Maayer et al., 2014). Studies with cold adapted microorganism are important due to their biotechnological potential (Cavicchioli et al., 2011), for improving our knowledge of how life thrives at low temperatures, and our understanding of how they impact global chemical cycles (Dedysh, 2011; De Maayer et al., 2014). Among such cold-environments, Antarctica stands out as the largest desert in the world, an environment with potential to render microbes that can vastly improve our knowledge of how life thrives in such harsh conditions and that can be considered a good analog to extraterrestrial environments, like Mars (Musilova et al., 2015; Goordial et al., 2016).

Here we report the isolation of uncultured microorganisms from a soil sample from Antarctica using a combination of simple techniques (Janssen et al., 2002; Sait et al., 2002; Davis et al., 2005; Tamaki et al., 2009; George et al., 2011; Tanaka et al., 2014). Slow growing bacteria were selected on diluted Nutrient Broth media solidified with Gellan gum after extended incubation periods (15 weeks) at a low temperature (12°C). Bacterial colonies on the isolation plates were observed under a stereo microscope, and colonies that appeared only after 4 weeks of incubation were selected, to obtain only slow-growing bacteria. Colonies were chosen based on their morphological characteristics and picking similar colonies that developed on the plates at the same time was avoided.

## Materials and Methods

### Sample Collection

**FIGURE 1:**
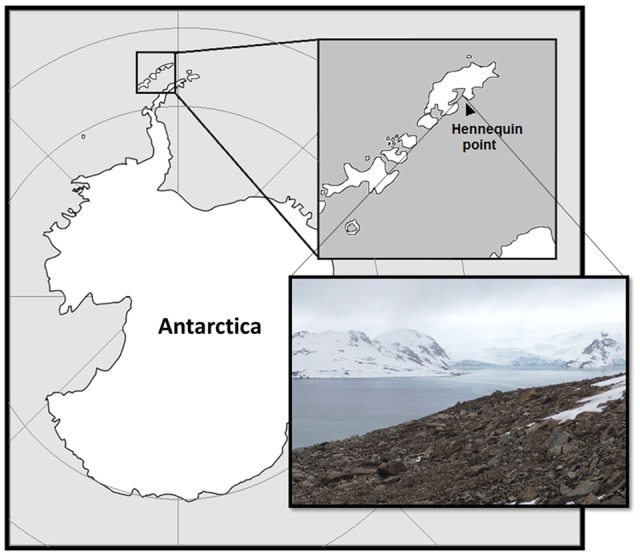
Sampling site at King George Island, Antarctica.

Samples were collected on December 29, 2014, at Hennequin Point, Admiralty Bay, King George Island, Antarctica (**Figure 1**) during the XXXIII Brazilian Antarctic Operation (2014/2015). Specific coordinates from the sampling site are 62°07.112′S, 58°23.199′W, 155 m altitude. The temperature during the sample collecting was around 0°C. Between 20 and 50 grams of soil was collected using flame-sterilized tools, stored in sterile tubes sealed in “Whirl-Pak” bags (Nasco, United States), and kept at -20°C during transportation, until the arrival in the laboratory in Brazil, where it was maintained at -20°C until isolation.

### Isolation Procedures

All contamination-sensitive procedures were performed inside a laminar flow hood. For isolation of the microorganisms, 2 g of soil were suspended in 10 mL of sterile 0.9% (w/v) NaCl solution and vortexed for 30 s. The supernatant was serially diluted (10^-1^ to 10^-6^) and spread in 1/100 diluted (0.08 g per liter) Difco Nutrient Broth – NB (Becton Dickinson, United States) plates (1/100 NB), solidified using 0.7% (w/v) Gellan gum (CP Kelco, United States) with the addition of MgSO_4_⋅7H_2_O (0.1% w/v) as a divalent cation to promote solidification; 8–10 plates for each dilution were prepared. Plates were then incubated at 12°C in the absence of light for up to 15 weeks. To avoid desiccation, plates were incubated inside polyethylene bags (Davis et al., 2011). Colony formation was followed weekly under a stereo microscope (up to ×40 magnification) and new colonies were marked as they appeared. Selection of the isolates was performed by picking only colonies that appeared after 4 weeks of incubation. Colonies were chosen based on their morphological characteristics, therefore picking similar colonies that developed on the plates at the same time was avoided. For isolation, the chosen colonies were streaked on new 1/100 NB plates. After isolation, cells were re-streaked on 1/100 NB plates, 1/10 NB plates (10 times less diluted), and Difco Reasoner’s 2A agar – R2A (Becton Dickinson, United States) for evaluating growth preferences. Whenever possible, cells were grown in liquid 1/100 NB, 1/10 NB, or R2A broth for generating biomass for cryostock preparation. Many of the isolates, however, did not developed well on liquid media. In these cases, biomass was collected from colonies in petri dishes. Stocks were prepared with 30% v/v glycerol and maintained at -80°C. All isolates are listed in Supplementary Table 1.

### DNA Extraction and 16S Sequencing

Genomic DNA was extracted using a PureLink Genomic DNA Mini Kit (Invitrogen, United States). 16S rRNA genes were amplified (initial denaturation step at 98°C for 5 min, followed by 30 cycles at 95°C for 30 s, 50°C for 30 s and 72°C for 2 min, followed by a final step at 72°C for 3 min) using the universal primers 27F and 1401R, purified using the GeneJet PCR purification kit (Thermo Scientific, United States), and sequenced using the BigDye Terminator kit (Applied Biosystems, United States), using the 27F, 1401R, and 518uF primers. Sequences were obtained by Sanger sequencing with an Abi Prism 3130xl Genetic Analyzer (Applied Biosystems, United States). Near the full sequence (1400 base pairs) of 16S rRNA was obtained. Sequences were deposited in Genbank under the access codes (KX990222.1 to KX990262.1). Phylogenetic trees were built using MEGA6 (Tamura et al., 2013), sequence alignments were performed with Muscle, and tree estimates were obtained using the maximum likelihood test (500 bootstraps). For sequence acquirement and analysis, EzTaxon (Kim et al., 2012) was used to obtain the top hits including type material sequences (described species). For environmental and cultured bacteria (non-type material sequences), Megablast (Genbank) top hits were used. In addition to EzTaxon and the NCBI database, our isolate’s sequences were also analyzed using the Silva database Project (Quast et al., 2013).

### Fluorescence Microscopy

For fluorescence microscopy imaging, cells were collected directly from plates and suspended in 50 μl of sterile 0.9% (w/v) NaCl solution. For cell membrane staining, the fluorescent dye FM1-43 (Invitrogen, United States) was used at a final concentration of 50 μg.mL^-1^. For DNA staining, the dye DAPI (Thermo Fisher, United States) was used at a final concentration of 15 μg.mL^-1^. For images, cells were placed over agar-pads [0.9% (w/v) NaCl solution solidified with 1.5% w/v agarose] and observed on a Nikon Eclipse TiE microscope (Nikon, Japan) equipped with a Plan APO VC Nikon 100× objective (NA = 1.4), a 25-mm SmartShutter, and Andor EMCCD i-Xon camera.

### Raman Spectroscopy and Pigments Characterization

For pigment evaluation, the isolates were analyzed by Raman spectroscopy, using a Renishaw (Renishaw PLC, Wotton-under-Edge, United Kingdom) inVia micro-Raman Spectrometer with laser line (532 nm), 50× objective, and CCD detector, and a Horiba-Jobin-Yvon micro-Raman Spectrometer with triple monochromator, laser line at 457 nm, 100× objective and CCD detector. The isolates were analyzed without further sample preparation, by removing the colonies from plates and depositing them onto glass slides. For *Aphanothece stagnina* and *Halobacterium salinarum* NRC-1 spectrum, cells were collected from liquid growth (BG11 media for *Aphanothece* and ATCC 217 medium for *Halobacterium*) centrifuged for 2 min at 5000 × *g*, the supernatant was discarded, and the pellet was deposited on glass slides. Low laser power was used to avoid thermal or photochemical damage. The acquired spectra were analyzed using the software Fityk (Wojdyr, 2010) and peak positions were estimated after peak fitting.

## Results and Discussion

### Microorganisms Belonging to Rare or Recently Described Genera Were Isolated during the First 8 Weeks of Incubation

Our isolation procedures focused on cold-tolerant, slow-growing microorganisms. With that in mind, we concentrated our efforts on colonies that developed on diluted medium plates after 4 weeks of incubation at 12°C. Since our objective was not to perform an ecological or abundance characterization of the sample, the fast-growing bacterial community present was not deeply studied in this work. In the same way, we did not collect all slow-growing colonies that appeared on the plates, as several similar slow-growing morphotypes were observed after the same incubation period. However, we observed that during the first weeks most of the bacteria that grew belonged to the genus *Arthrobacter* and *Sphingomonas*, inferred by 16S rRNA sequencing (data not shown), as well as several colonies that visually appeared to be fungi and yeasts, which were confirmed by observation under a microscope.

Ultimately, we selected 41 morphologically distinct bacterial colonies that grew in the period of 4–14 weeks of incubation (Supplementary Table 1). Considering the top-hit 16S rRNA sequence matches of our set of slow-growing isolates, it was possible to classify them in at least 26 distinct genera: *Sphingomonas, Phycicoccus, Quadrisphaera, Friedmanniella, Methylobacterium, Lapillicoccus, Polaromonas, Motilibacter, Rhodoferax, Kineosporia, Nocardioides, Flavitalea, Polymorphobacter, Hymenobacter, Ryzobacter, Nakamurella, Angustibacter, Bradyrhizobium*, and *Marmoricola.* Ten isolates scored highest similarity within the genera *Paracraurococcus, Rhodopila, Acidothermus, Frankia, Beijerinckia, Modestobacter*, and *Conexibacter*, although for these isolates, due to the low 16S rRNA sequence similarity and their position in our phylogenetic trees, they all likely belong to undescribed genera. Although there are established “cutoff” values for determining the novelty of an isolate based on 16S rRNA sequence similarity, there is no consensus for this value (Janda and Abbott, 2007). Conservative thresholds consider novel species when 16S rRNA sequence similarity is less than 97%, novel genera and families when these values are between 95 and 91%, and novel order when the values are below 91% (Nichols et al., 2010; Rossi-Tamisier et al., 2015). However, more recent studies propose cutoff values of 98.7% for novel species (Stackebrandt and Ebers, 2006). In addition, as recently pointed out by Rossi-Tamisier et al. (2015), classification for novel genera and families must also consider minimum and maximum similarity values observed among species already described for that particular group (Rossi-Tamisier et al., 2015), since there is large variation between different groups of bacteria. For this manuscript discussion, the conservative cutoff values will be considered, as well as the position on phylogenetic trees.

Our methodology was successful in obtaining novel, previously uncultured microorganisms from an Antarctic soil sample. Microorganisms belonging to rare or recently described genera (**Table 1**), for example *Lapillicoccus, Flavitalea, Quadrisphaera, Motilibacter*, and *Polymorphobacter* were obtained, as determined by phylogenetic trees (Supplementary Figures 1A–E).

**Table 1:** Isolates belonging to rarely isolated genera or potentially undescribed generas and families.

**Isolate code**	**Weeks to isolation**	**Accession code**	**Highest 16S rRNA sequence similarity** **(Type strain-Eztaxon)**	**Highest 16S rRNA sequence similarity** **(Genbank)**
Ap07E	5	KX990227.1	*Quadrisphaera granulorum* AG019 (98.2%) – [AY831385]	*Quadrisphaera granulorum* strain CS4 (99%) – [AM887695.2]
Ap15E	4	KX990235.1	*Motilibacter peucedani* RP-AC37 (97.46%) – [FM998003]	*Actinomycetales bacterium* OS1-23 (98%) – [FN649461.1]
Ap13E	5	KX990233.1	*Lapillicoccus jejuensis* R-Ac013 (98.4%) – [AM398397]	*Lapillicoccus jejuensis* R-Ac013 (98%) – [AM398397]
Ap19E	7	KX990239.1	*Flavitalea populi* HY-50R (96.72%) – [HM130561]	Uncultured *Flavitalea* sp. clone SNNP_2012-54 (99%) – [JX114387.1]
Ap23E	8	KX990242.1	*Polymorphobacter multimanifer* 272-7 (95.66%) – [AB649056]	Uncultured alpha proteobacterium clone IC4022 (99%) - [HQ622730.1]
Ap32E	8	KX990250.1	*Paracraurococcus ruber* NS89 (95.62%) – [D85827]	Uncultured endolithic bacterium clone SM_01_BAC (99%) – [AB473915.1]
Ap20E	8	KX990240.1	*Lapillicoccus jejuensis* R-Ac013 (98.12%) – [AM398397]	Uncultured *Lochheadia* sp. clone Plot4-B02 (98%) – [EU449557.1]
Ap25E	9	KX990244.1	*Frankia alni* ACN14A (93.35%) – [CT573213]	Uncultured bacterium clone MA03C08 (98%) – [FM873556.1]
Ap29E	9	KX990248.1	*Rhodopila globiformis* DSM 161 (93.49%) – [D86513]	Uncultured alpha proteobacterium clone IC4004 (96%) – [HQ622721.1]
Ap42E	10	KX990257.1	*Acidothermus cellulolyticus* ATCC 43068 (92.08%) – [CP000481]	Uncultured bacterium clone ncd260b10c1 (97%) – [HM270084.1]
Ap46E	11	KX990261.1	*Frankia alni* ACN14A (93.39%) – [CT573213]	Uncultured bacterium clone MA03C08 (98%) – [FM873556.1]
Ap43E	12	KX990258.1	*Rhodopila globiformis* DSM 161 (92.55%) – [D86513]	Uncultured bacterium clone Bas-7-52 (97%) – [GQ495410.1]
Ap44E	12	KX990259.1	*Modestobacter versicolor* CP153-2 (94.5%) – [AJ871304]	Uncultured bacterium clone 3-952 (98%) – [KC554168]
Ap36E	13	KX990253.1	*Beijerinckia derxii* subsp. venezuelae DSM 2329 (94.28%) – [AJ563934]	Uncultured bacterium clone 1174-901-15 (98%) – [AB128887.1]
Ap38E	13	KX990255.1	*Conexibacter woesei* DSM 14684 (93.7%) – [CP001854]	Rubrobacteridae bacterium Gsoil 1167 (99%) – [AB245333.1]
Ap45E	14	KX990260.1	*Conexibacter arvalis* KV-962 *(94.46%)* – [AB597950]	Uncultured actinobacterium clone UMAB-cl-13 (98%) – [FN811197.1]

The genus *Flavitalea* was originally described in 2011 (Wang et al., 2011) and has three species described to date. Our isolate Ap19E clustered together in our phylogenetic tree with *F. populi* and *F. gansuensis* (Supplementary Figure 1B), which suggest that these organisms belong to the same genus. The genus *Lapillicoccus* was first described in 2007 (Lee and Lee, 2007). The type-species *Lapillicoccus jejuensis* R-Ac013 is the only currently described species within the genus. We isolated two morphologically distinct bacteria (Ap13E and Ap20E) that, because of the 16S rRNA sequence similarity and position within the phylogenetic tree (**Table 1** and Supplementary Figure 1A), probably belongs to the *Lapillicoccus* genus.

The type strain *Q. granulorum* AG019 was described in 2005 (Maszenan, 2005), and it is the only described species of the genus. Our isolate Ap07E formed an independent branch together with *Q., granulorum* AG019 (Supplementary Figure 1C), Additional assays performed with our isolate demonstrated that the Ap07E strain is capable of growing at 4°C and does not develop at temperatures of 30°C or higher (data not shown), whereas *Q. granulorum* AG019 has been reported as not capable of growing at temperatures below 15°C, and with optimum growth temperature of 37°C (Maszenan, 2005).

*Polymorphobacter multimanifer* was first described in 2014 (Fukuda et al., 2014) and the genus has two described species to date. Our isolate Ap23E grouped together with these two species in our phylogenetic tree (Supplementary Figure 1D), however, due to the low 16S rRNA sequence similarity with the closest type strain (95.66% similarity with *Polymorphobacter multimanifer* 272-7)it is quite possible that our isolate Ap23E might constitute a new *Polymorphobacter* species. Finally, the species *Motilibacter peucedani* was described in 2012 (Lee, 2012) and a subsequent publication describing a second species, *Motilibacter rhizosphaerae*, also proposed it as a new genus and new family of the suborder *Frankinae*, family *Motilibacteraceae* (Lee, 2013). Due to the position within the phylogenetic tree (Supplementary Figure 1E) we suggest that our isolate Ap15E belongs to the same genus and family.

### Longer Incubation Periods Allowed Isolation of Members of Potentially Novel Bacterial Genera and Families

In addition, of our slow growing set of isolates (Supplementary Table 1), we obtained organisms with 16S rRNA sequence similarity ranging from 92.08 to 94.6% to any other known cultured species (**Table 1**; Isolates Ap25E, Ap29E, Ap36E, Ap38E Ap42E, Ap43E, Ap44E, Ap45E, and Ap46E). These isolates were obtained after 8 to 14 weeks of incubation. Particularly for these isolates, microscopic observations of the plates were vital for isolation, since many of them developed as extremely small colonies and were surrounded by faster growing bacterial colonies and filamentous fungi that could eventually overgrow them.

The isolates Ap29E, Ap32E, Ap36E and Ap43 were considered Proteobacteria. They all share low 16S rRNA sequence similarity (∼94%) with the closest described species (**Table 1**) and with microbes belonging to non-type material (94–95%) except for Ap32E. This isolate, Ap32E, clustered with a sequence of *Acetobacteraceae* bacterium WS10 in our phylogenetic tree (Supplementary Figure 2A). Recently, Kim et al. (2016) have proposed the isolate WS10 as *Dankookia rubra* gen. nov. It is possible therefore, after confirmation of these new genera that our isolate will belong to the *Dankookia* genus, as it shares 98% sequence similarity with the WS10 strain.

**FIGURE 2:**
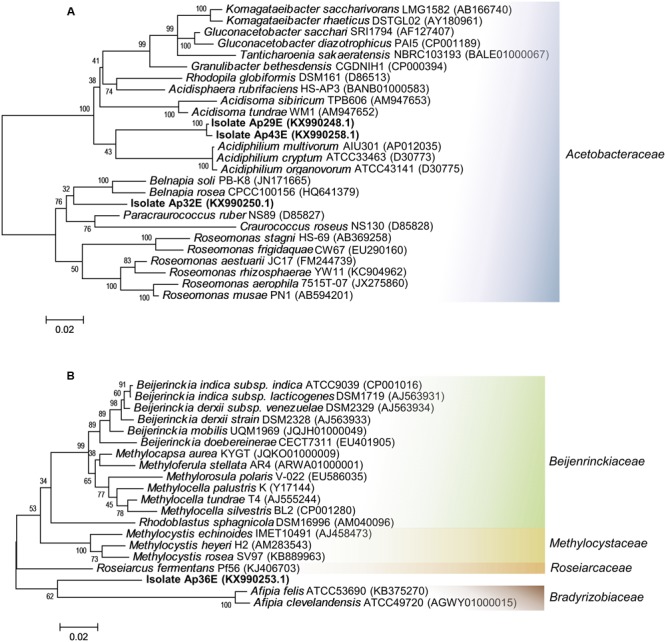
Maximum likelihood phylogenetic trees of the isolates Ap29E, Ap32E, Ap36E and Ap43E. **(A)** Phylogenetic affiliation of the isolates Ap29E, Ap32E and Ap43E and the most similar 16S sequences of type material organisms. **(B)** Phylogenetic affiliation of the isolates Ap36E and the most similar 16S sequences of type material organisms. Scale bar indicates a change of 0.02 per nucleotide.

The isolates Ap32E, Ap29E, Ap43E belong to the big bacterial family *Acetobacteraceae* (**Figure 2A**). Ap29E and Ap43E share 99% 16S rRNA sequence similarity and have similar phenotypical characteristics, suggesting these two isolates belong to the same species. The isolate Ap36E grouped with bacteria from the order *Rhizobiales* (**Figure 2B**). However, due to their low 16S rRNA sequence similarity, they did not appear to cluster into any described genus (Ap29E and Ap43E) or family (Ap36E) in our phylogenetic trees. By constructing a tree with the addition of environmental sequences and cultured bacteria, Ap29E and Ap43E cluster together with several sequences belonging to cold environments, glacier ice, snow, and ice cores, possibly indicating a cold and dry adapted, but yet undescribed genus (Supplementary Figure 2A), whereas Ap36E clustered together with environmental sequences found in both cold and warm environments including marine sediments, glaciers, snow, and gastrointestinal samples (Supplementary Figure 2D). Such sequences, together with our isolate, form an independent branch from the closest families.

**FIGURE 3:**
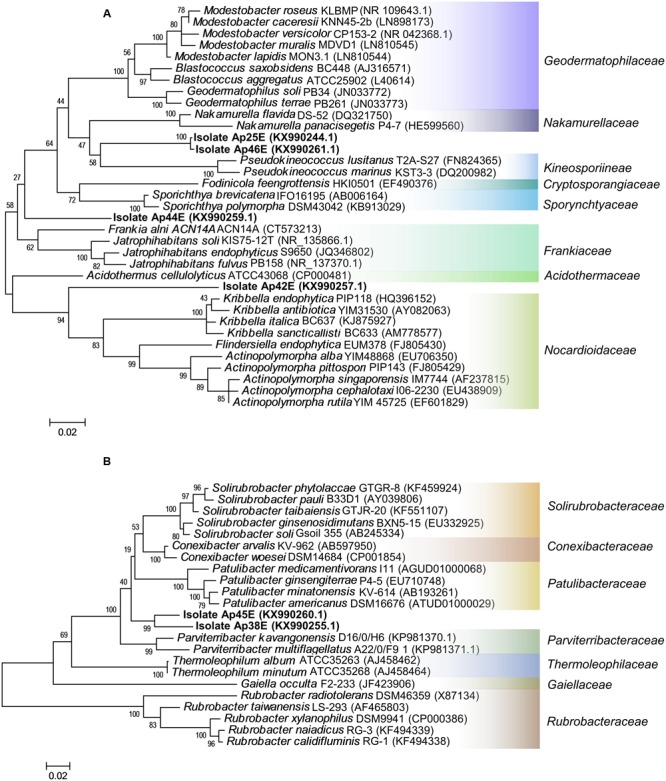
Maximum likelihood phylogenetic trees of the isolates Ap25E, Ap38E, Ap42E, Ap44E, Ap45E, Ap46E. **(A)** Phylogenetic affiliation of the isolates Ap25E, Ap42E, Ap44E and Ap46E and the most similar 16S sequences of type material organisms. **(B)** Phylogenetic affiliation of the isolates Ap38E, and Ap45E, and the most similar 16S sequences of type material organisms. Scale bar indicates a change of 0.02 per nucleotide.

The isolates Ap25E, Ap42E, Ap44E, and Ap46E belong to the order *Actinomycetales*. They all share low 16S rRNA sequence similarity with any described species (**Table 1**), some of them as low as 92%. By constructing a phylogenetic tree with the highest 16S rRNA sequence similarities (type material sequences), we could not group them into any described genus or family (**Figure 3A**). None of these organisms share 16S rRNA sequence similarity higher than 94% with any cultured bacteria (non-type material, evaluated using Megablast). As a highlight, Ap25E and Ap46E isolates clustered together in a large group, containing several sequences belonging to mountain environments (Tang et al., 2016) and desert environments (Supplementary Figure 2B). Three of such sequences were reported at the Yungay region, a hyperarid region at the Atacama desert (Connon et al., 2007), and the authors also proposed such sequences as belonging to a novel, undescribed genera. Curiously, the authors attempted to isolate bacteria from such samples, but did not manage to retrieve isolates from the cluster, after 3 weeks of incubation.

The Yungay environment has extreme low water activity, which could force microbes to replicate during rare precipitation events that might occur (Connon et al., 2007). In this scenario, the slow growth behavior of our isolates both during and after isolation could even represent an environmental adaptation of this bacterial cluster to such an environment and might be the reason that no representatives of such group had been isolated until now. Considering that our isolates Ap25E and Ap46E are the first cultured representatives from this cluster of bacteria, their characteristics may be useful for extrapolating this adaptive behavior to other deserts, such as the Atacama, as well help in further isolation attempts.

Finally, the isolates Ap38E and Ap45E grouped together with bacteria belonging to a deep branching phylogenetic lineage within the phylum *Actinobacteria*, class *Thermoleophilia*, order *Solirubrobacterales* (**Figure 3B**), sharing ∼94% 16S rRNA sequence similarity with other described species (**Table 1**). Sequences of organisms belonging to class *Thermoleophilia* have been constantly reported in samples from Antarctica (Chong et al., 2012; Aislabie et al., 2013; Kumar et al., 2013; Rampelotto et al., 2015; Ji et al., 2016; Pudasaini et al., 2017), reaching relative abundances of 15% in some samples (Ji et al., 2016). Although highly present, no isolate of this class has been reported from this type of environment to date, making this the first report of *Thermoleophilia* isolation from an Antarctic sample in addition to the novelty of this species. We observed that within 80 days, both isolates (Ap38E and Ap45E) grew at 10 and 15°C, whereas no growth was observed at 30°C. At 25°C, only Ap38E presented weak growth, but less than that observed at 15 and 10°C (Supplementary Figure 3). This indicates that these isolates can be potential psychrophilic microorganisms, an important feature considering that all described species within the order *Solirubrobacterales* are mesophilic (Whitman and Suzuki, 2015; Foesel et al., 2016).

The phylogenetic tree (using type material sequences) did not place our isolates (Ap38E and Ap45E) in any described genera or family within the order *Solirubrobacterales*. Our isolates grouped together and formed a distinct branch near the families *Conexibacteraceae, Patulibacteraceae, Solirubrobacteraceae* and the recently described *Parviterribacteraceae* (Foesel et al., 2016), as can be seen in **Figure 3B**. Although grouped together in an independent branch, Ap38E and Ap45E share only 95% of 16S rRNA sequence similarity between each other, suggesting they are two different species within the same family.

By including the top hits of environmental sequences and cultured but non-type material in the phylogenetic tree (Supplementary Figure 2C) we observed that our isolates form a group that cluster with environmental sequences from multiple geographic locations all subjected to great UV influx and/or low water activity: Antarctica (Chong et al., 2012), mineral surface biofilms (Ragon et al., 2012), Tibetan soil, and the Llullaillaco volcano (>6000 m) and the Atacama Desert (Lynch et al., 2012), which can suggest a highly adapted group of bacteria to such extreme conditions. Interestingly, our isolate Ap38E also clustered together with two sequences: *Rubrobacteridae* bacterium Gsoil 319 (accession code AB245332) and *Rubrobacteridae* bacterium Gsoil 1167 (accession code AB245333) sharing 99% 16S rRNA sequence similarity with both of these sequences. The isolate Ap45 on the other hand, shares only 95% similarity with these two sequences. However, both the *Rubrobacteridae* bacterium Gsoil 319 and *Rubrobacteridae* bacterium Gsoil 1167 sequences were deposited in GenBank in 2006, but have not been associated with any publication until now. Despite the information that both organisms seem to have been isolated from a ginseng field (available on GenBank), there is no additional information regarding such isolates, which makes any possible comparison with our two isolates difficult. Therefore, our isolates have a high potential to constitute two different novel, distinct species within the order *Solirubrobacterales*.

**FIGURE 4:**
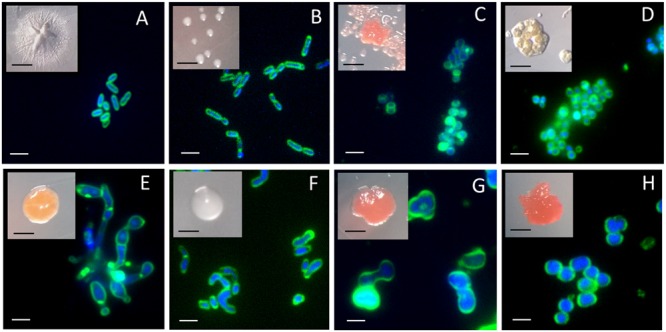
Colonies and fluorescence microscopy images of isolates obtained from the Hennequin point sample on diluted NB media. **(A)** Isolate Ap38E; **(B)** Isolate Ap45E; **(C)** Isolate Ap25E. Similar images were obtained for isolate Ap46E and therefore are not shown; **(D)** Isolate Ap42E; **(E)** Isolate Ap44E; **(F)** Isolate Ap36E; **(G)** Isolate Ap29E. Similar images were obtained with isolate Ap43E and therefore are not shown; **(H)** Isolate Ap32E. Membrane were stained with FM1-43 and DNA were stained with DAPI. Black scale bars: 1 mm. White scale bars: 2 μm.

In fact, our isolates Ap38E and Ap45E have distinct colony morphologies, but both isolates are small rods of 0.6–1.2 μm (**Figures 4A,B**), similar in size with the bacteria to the family *Conexibacteraceae* (Monciardini, 2003; Seki et al., 2012). The isolates Ap25E, Ap46E, and Ap42E are also composed of small cells, however, they are coccoid in shape and grow in aggregates (**Figures 4C,D**). To note, the isolates Ap25E and Ap46E, which clustered with several sequences found in desert and mountain soils, had strong red pigmentation. The isolate Ap44E grows as orange colored colonies, and its cells are filamentous and aggregated (**Figure 4E**). The cell morphology of isolate Ap36E is curious: it appears to be dimorphic and undergo asymmetric septation (**Figure 4F**). Such features have already been described for other *Rhizobiales* bacteria (Brown et al., 2012). Isolates Ap29E, Ap32E, and Ap43E form colonies with intense red color (**Figures 4G,H**). Microscopic observations showed that they are all coccoid cells (∼2 μm).

**FIGURE 5:**
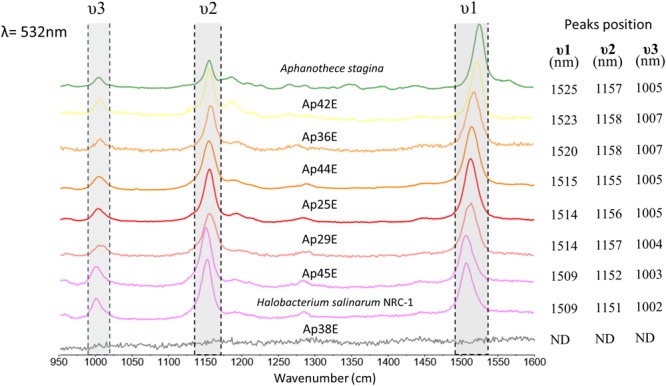
Raman spectra of the isolated colonies with excitation at 532 nm with the most intense Raman bands of carotenoids.

Pigments were characterized using Raman spectroscopy (**Figure 5**). Carotenoids cause very intense Raman bands, characterized by three typical peaks, at 1520, 1150, and 1000 cm^-1^ (de Oliveira et al., 2010; Jehlička et al., 2014a). With the exception of isolate Ap38E, carotenoids were observed in all other isolates. Surprisingly, Ap45E and Ap36E also produced carotenoids under our growth conditions, despite not having visible colony coloration (**Figures 4B,F**). To highlight, we observed that the Raman spectra acquired for isolate Ap45E (which forms light-pink colonies) is remarkably similar to the spectra acquired for the *Halobacterium salinarum* NCR-1, a microorganism for which the Raman signal is due to bacterioruberin, represented by low stretching values for all peaks, when compared with other carotenoids (Jehlička et al., 2014b). Bacterioruberin is an unusual C50 carotenoid constantly studied and analyzed by Raman spectroscopy (Imperi et al., 2007; Morillas et al., 2015), and present in some halophilic archaea and some actinobacteria belonging to the family *Rubro bacteraceae* (Saito et al., 1994; Imperi et al., 2007).

We sought to detect presence of bacteriochlorophyll, especially in isolates Ap29E and Ap43E, which are clustered near the *Acidiphilium* genera in our phylogenetic tree, a group that has been shown to produce bacteriochlorophyll (Kishimoto et al., 1995). However, we failed to detect characteristic peaks (750, 910, and 1630 cm^-1^) for the pigment (Donohoe et al., 1988; Jehlička et al., 2014a) for Ap29E in our growth conditions, even when using different Raman lasers with the best resonance for chlorophyll and bacteriochlorophyll (Supplementary Figure 4).

More concentrated medium (NB 1/10) slightly improved cell growth for most of the isolates. However, with the exception of isolate Ap32E, the isolates Ap25E, Ap29E, Ap36E, Ap38E, Ap42E, Ap43E, Ap44E, Ap45E, and Ap46E failed to grow on R2A medium. R2A is commonly employed for bacterial isolation from Antarctica and similarly cold environmental samples (Skidmore et al., 2000; Aislabie et al., 2006, 2013; Clocksin et al., 2007; Steven et al., 2007; Vester et al., 2013; Grzesiak et al., 2015), as it is considered a low nutrient medium known to yield a high number of isolates (Reasoner and Geldreich, 1985). Most of our rare isolates failed to develop on R2A, and therefore would have been missed if just R2A had been employed in this study.

We decided to investigate more deeply four isolates (Ap25E, Ap38E, Ap42E, and Ap45E), regarding their growth preferences, using a combination of pure and diluted media, neutral (pH 7) and more acidic pH (pH 5) NB media (5 and 100%), TSA media (5 and 100%) and R2 media solidified with Gellan gum (10 and 100%) (Supplementary Table 2). Interestingly, all tested isolates showed a preference trend toward neutral, diluted media; for some cases (Isolates Ap38E, Ap25E, and Ap45E), growth was only recorded on diluted media, suggesting that these organisms are indeed oligotrophic. In addition, it was not possible to improve growth speed of the isolates, at least in our tested conditions: isolates Ap25E and Ap45E still kept their slow-growth behavior.

As a highlight, using Gellan-gum as solidifying agent, we have managed to grow isolates Ap42E and Ap38E on R2 media. Ap38E developed well at diluted R2 media (10%), however, it has not grown well on pure, 100% R2 media, suggesting that this medium does not achieve the oligotrophic requirements for this isolate (Supplementary Table 2). Once again, Ap42E and Ap38E failed to grow on R2A media (R2 solidified with Agarose), even when using colonies that developed on R2 media solidified with Gellan gum as inoculum.

Overall, our data demonstrate that is possible to retrieve recalcitrant bacteria from Antarctic samples using long-incubation periods, oligotrophic media, low temperatures, and selecting for slow-growing bacteria. Such a strategy allowed us to retrieve several bacteria with low 16S rRNA sequence similarity to any described isolate. However, several of such isolates clustered together not only with Antarctic samples, but also with sequences found in other cold, extreme habitats, such as glaciers, ice-cores, snow, mountain soil, epilithic biofilms and other deserts, like the Atacama and Taklamakan. As a highlight, we isolated two distinct, cold-adapted species belonging to the class *Thermoleophilia*, order *Solirubrobacterales* from Antarctica that due to their low 16S rRNA sequence similarity with other described species, might belong to an undescribed genera or family. Currently, we are improving growth conditions of our isolates, which will allow the expansion of knowledge about slow-growing microbial organisms in cold-environments, their environmental roles, stress resilience, as well as increase taxonomic data.

## Author Contributions

AP, AB, AF, and FR executed experiments and performed analysis of the data. AP and FR conceived the original idea and designed experiments. FR, DG, and VP conducted the samples’ collection and transportation. All authors contributed to critical interpretation of the data, writing and revising the manuscript. All authors approved the final version manuscript.

## Conflict of Interest Statement

The authors declare that the research was conducted in the absence of any commercial or financial relationships that could be construed as a potential conflict of interest.
