# Supplementary material for: Isolation of Uncultured Bacteria from Antarctica Using Long Incubation Periods and Low Nutritional Media
Source: Front Microbiol. 2017 Jul 14;8:1346. doi: 10.3389/fmicb.2017.01346 (PMC5509766; doi:10.3389/fmicb.2017.01346)
Supplement: Supplementary file 1 [file Data_Sheet_1.pdf]

# Supplementary Material

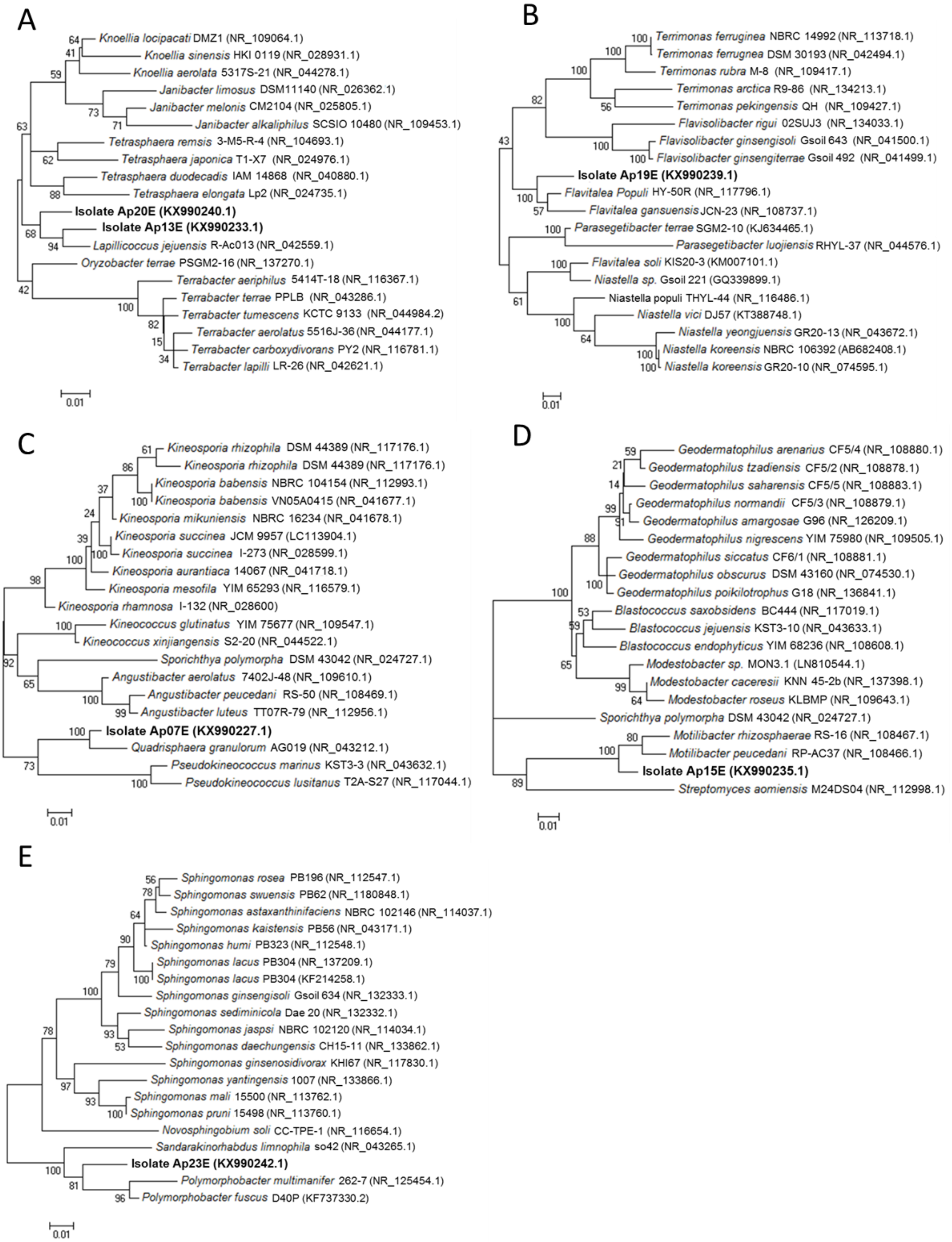


**Supplementary Figure 1.** Phylogenetic affiliation of the isolates Ap20E, Ap13E, Ap19E, Ap07E, Ap23E and Ap15E and most similar type material 16S sequences. (A) Ap20E and Ap13E; (B) Ap19E; (C) Ap07E; (D) Ap23E; (E) Ap15E

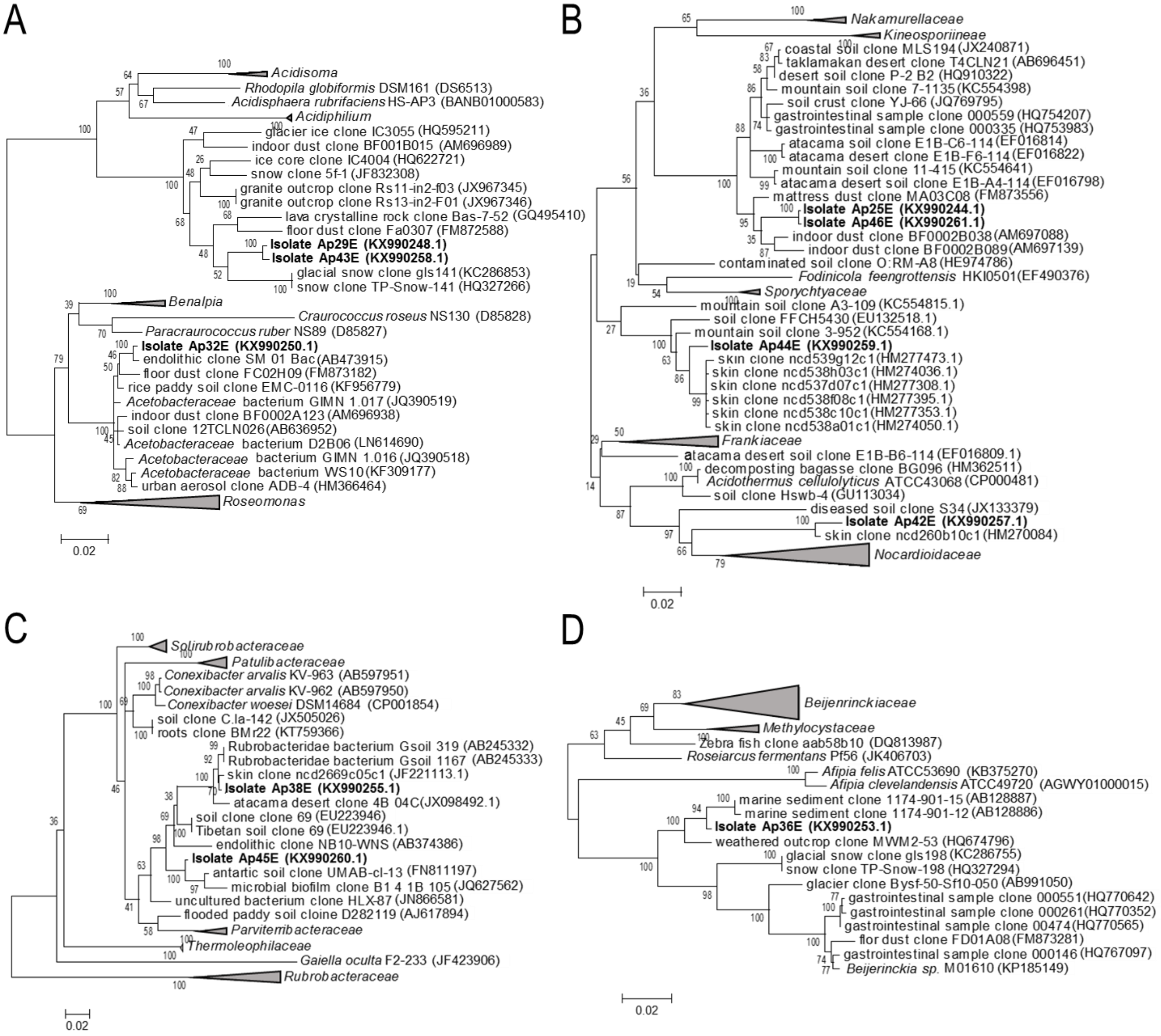


**Supplementary Figure 2.** Phylogenetic trees of the isolates Ap25E, Ap29E, Ap32E, Ap36E, Ap38E, Ap42E, Ap,43E, Ap44E, Ap45E, Ap46E, considering high similarity sequences considering both cultured and environmental data. (A) Phylogenetic affiliation of the isolates Ap29E, Ap32E, Ap43E and the most similar 16S sequences. (B) Phylogenetic affiliation of the isolates Ap25E, Ap42E, Ap44E and Ap46E and most similar 16S sequences. (C) Phylogenetic affiliation of the isolates Ap38E, Ap45E and most similar 16S sequences. (D) Phylogenetic affiliation of the isolates Ap38E, Ap45E and most similar 16S sequences. The scale bar represents a change of 0.02 per nucleotide.

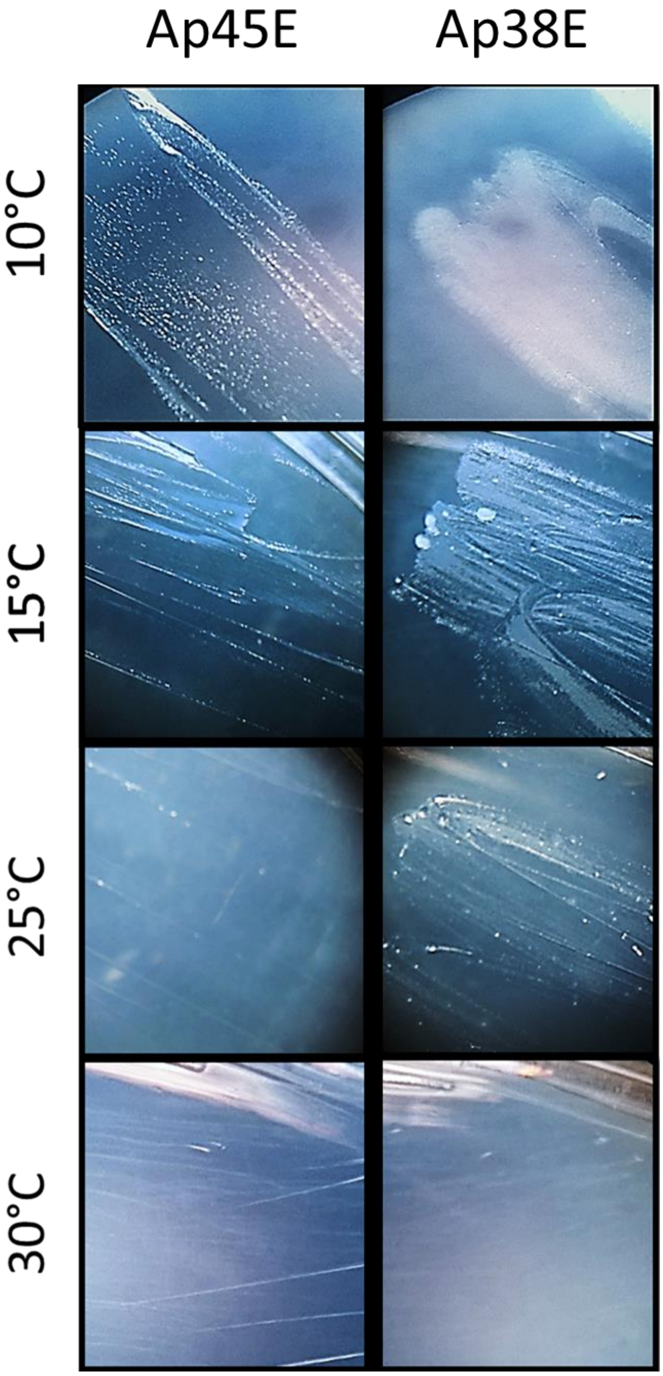


**Supplementary Figure 3**. Growth at different temperatures for the isolates Ap45E and Ap38E, on DNB 10% media after 80 days of incubation. As can be observed, growth occurs at 10°C and 15°C, whereas little or no growth is observed at higher temperatures. At 25°C weak growth is observed for Ap38E, whereas no growth was observed for Ap45E. At 30°C, no growth was detected for both isolates.

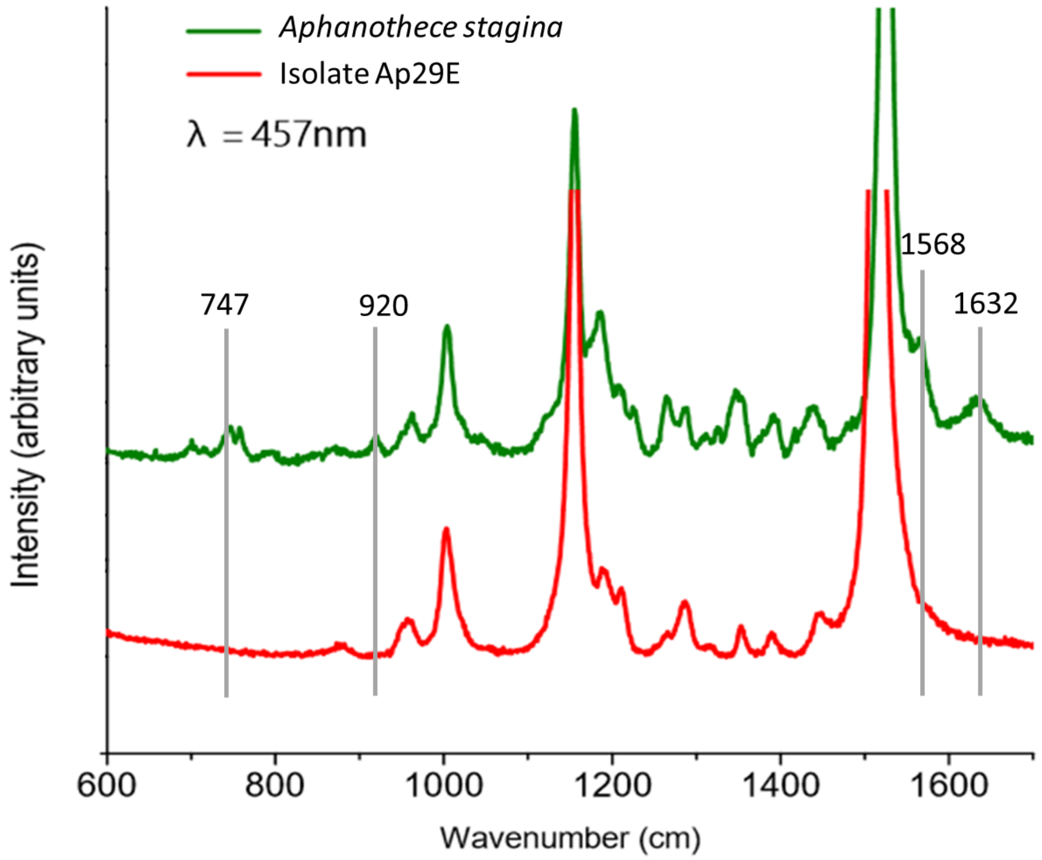


**Supplementary Figure 4**. Raman spectra acquired with the blue laser (457nm) of the isolate Ap29E and *Aphanothece stagina*.

**Supplementary Table 1.** Slow-growing isolates obtained using 1/100 NB Gellan gum plates

| Isolate code | Weeks until isolation | Accession code | Highest 16S sequence similarity (Cultured - Ezbiotaxon) | Highest 16S sequence similarity (Genbank) |
|---|---|---|---|---|
| Ap02E | 4 | KX990222.1 | *Sphingomonas molluscorum* **(99.78%) –** [AB248285] | *Sphingomonas* sp. PA223 **(99%)** – [AM900786.1] |
| Ap03E | 4 | KX990223.1 | *Sphingomonas mali* IFO 15500 **(97.7%)** – [Y09638.1] | *Sphingomonas sp.* R-36505 **(98%)** - [FR682707.1] |
| Ap04E | 4 | KX990224.1 | *Phycicoccus ochangensis* l1b-b9 **(98.04%) –** [NR_132688.1] | Uncultured bacterium clone AK1DE1_08H **(99%) -** [GQ396990.1] |
| Ap05E | 4 | KX990225.1 | *Sphingomonas daechungensis* CH15-11 **(97.17%)** – [JQ772481] | Uncultured bacterium mus-a20 **(99%)** – [JN023506.1] |
| Ap06E | 4 | KX990226.1 | *Sphingomonas daechungensis* CH15-11 **(97.28%)** – [JQ772481] | Uncultured bacterium mus-a20 **(99%)** – [JN023506.1] |
| Ap07E | 5 | KX990227.1 | *Quadrisphaera granulorum* AG019 **(98.2%)** – [AY831385] | *Quadrisphaera granulorum* strain CS4 **(99%) –** [AM887695.2] |
| Ap08E | 5 | KX990228.1 | *Friedmanniella okinawensis* FB1 (**99.34%)** - [ NR_112796.1] | *Friedmanniella okinawensis* FB1 **(99%) –** [ NR_112796.1] |
| Ap09E | 4 | KX990229.1 | *Phycicoccus ochangensis* l1b-b9 **(97.9%) –** [NR_132688.1] | *Phycicoccus aerophilus* S32341 **(98%)** – [AB649009.1] |
| Ap10E | 4 | KX990230.1 | *Methylobacterium goesingense* iEII3 **(99.63%) –** [AY364020] | *Methylobacterium adhaesivum* z88a **(100%) -** [AB698722.1] |
| Ap11E | 4 | KX990231.1 | *Methylobacterium goesingense* iEII3 **(99.63%) –** [AY364020] | *Methylobacterium adhaesivum* z88a **(100%)** - [AB698722.1] |
| Ap12E | 7 | KX990232.1 | *Sphingomonas sediminicola* Dae 20 **(97.28%)** – [AB258386] | Uncultured bacterium clone ANTLV1_H10 **(99%) –** [DQ521501.1] |
| Ap13E | 5 | KX990233.1 | *Lapillicoccus jejuensis* R-Ac013 **(98.4%)** – [AM398397] | *Lapillicoccus jejuensis* R-Ac013 **(98%)** – [AM398397] |
| Ap14E | 5 | KX990234.1 | *Polaromonas vacuolata* 34-P **(98.2%) –** [U14585] | *Polaromonas* sp. R-36500 **(99%)** – [FR682711.1] |
| Ap15E | 4 | KX990235.1 | *Motilibacter peucedani* RP-AC37 **(97.46%)** – [FM998003] | *Actinomycetales bacterium* OS1-23 (9**8%)** – [FN649461.1] |
| Ap16E | 4 | KX990236.1 | *Albidiferax ferrireducens* T118 **(98.72%)** – [CP000267] | *Albidiferax* sp. R-37567 **(99%) –** [FR691423.1] |
| Ap17E | 8 | KX990237.1 | *Kineosporia rhamnosa* JCM 9954 **(99.05%)** – [AB003935] | *Kineosporia rhamnosa* strain I-132 **(99%)** – [NR_028600.1] |
| Ap18E | 6 | KX990238.1 | *Nocardioides deserti* SC8A-24 **(96.89%) –** [KM816582] | Nocardioides sp. R-36473 **(99%) –** [FR682687.1] |
| Ap19E | 7 | KX990239.1 | *Flavitalea populi* HY-50R **(96.72%) –** [HM130561] | Uncultured Flavitalea clone SNNP_2012-54 **(99%) –** [JX114387.1] |
| Ap20E | 8 | KX990240.1 | *Lapillicoccus jejuensis* R-Ac013 **(98.12%)** – [AM398397] | Uncultured Lochheadia sp. clone Plot4-B02 **(98%) –** [EU449557.1] |
| Ap21E | 6 | KX990241.1 | *Sphingomonas sediminicola* Dae 20 **(97.2%)** – [AB258386] | Uncultured bacterium clone ANTLV1_H10 **(99%)** – [DQ521501.1] |
| Ap23E | 8 | KX990242.1 | *Polymorphobacter multimanifer* 272-7 **(95.66%) -** [AB649056] | Uncultured alpha proteobacterium clone IC4022 **(99%)** - [HQ622730.1] |
| Ap24E | 6 | KX990243.1 | *Hymenobacter antarcticus* VUG-A42aa **(98.12%) –** [EU155012] | *Hymenobacter sp.* TMT1-67 (99%) – [JX949782.1] |
| Ap25E | 9 | KX990244.1 | *Frankia alni* ACN14A **(93.35%)** – [CT573213] | Uncultured bacterium clone MA03C08 **(98%)** – [FM873556.1] |
| Ap26E | 9 | KX990245.1 | *Nocardioides islandensis* MSL 26 **(96.36%) –** [EF466123] | Uncultured bacterium clone D3-119 **(97%) –** [KC554926.1] |
| Ap27E | 9 | KX990246.1 | *Sphingomonas oligophenolica* JCM 12082 **(99.06%)** – [AB018439] | Uncultured bacterium BF0002C010 **(99%) –** [AM697313.1] |
| Ap28E | 8 | KX990247.1 | *Rhizobacter fulvus* Gsoil 322 (**98.92%)** – [AB245356] | Uncultured bacterium clone JFJ-ICE-Bact-28 **(99%)** – [AJ867658.1] |
| Ap29E | 9 | KX990248.1 | *Rhodopila globiformis* DSM 161 **(93.49%)** – [D86513] | Uncultured alpha proteobacterium clone IC4004 **(96%)** – [HQ622721.1] |
| Ap31E | 7 | KX990249.1 | *Sphingomonas sediminicola* Dae 20 **(97.18%) -** [AB258386] | Uncultured bacterium clone ANTLV1_H10 **(99%)** – [DQ521501.1] |
| Ap32E | 8 | KX990250.1 | *Paracraurococcus ruber* NS89 **(95.62%)** – [D85827] | Uncultured endolithic bacterium clone SM_01_BAC **(99%)** – [AB473915.1] |

| | | | | |
|---|---|---|---|---|
| Ap33E | 6 | KX990251.1 | *Nakamurella panacisegetis* P4-7 **(98.92%) –** [HE599560 ] | *Nakamurella panacisegetis* P4-7 **(99%) –** [HE599560 ] |
| Ap35E | 8 | KX990252.1 | *Angustibacter luteus* TT07R-79 **(98.26%) –** [AB512285] | *Angustibacter luteus* strain AL8 **(98%) –** [KU258205.1] |
| Ap36E | 13 | KX990253.1 | *Beijerinckia derxii subsp. Venezuelae* DSM 2329 **(94.28%)** – [AJ563934] | Uncultured bacterium clone 1174-901-15 **(98%)** – [AB128887.1] |
| Ap37E | 9 | KX990254.1 | *Sphingomonas mali* IFO 15500 **(98.07%)** – [Y09638] | Uncultured *Sphingomonas* sp. clone DOL43 **(99%) –** [HM224424.1] |
| Ap38E | 13 | KX990255.1 | *Conexibacter woesei* DSM 14684 **(93.7%)** – [CP001854] | Rubrobacteridae bacterium Gsoil 1167 **(99%) –** [AB245333.1] |
| Ap41E | 13 | KX990256.1 | *Bradyrhizobium erythrophlei* CCBAU 53325 **(98.43%) –** [KF114645] | Uncultured bacterium clone B8-55 **(99%) –** [KF494604.1] |
| Ap42E | 10 | KX990257.1 | *Acidothermus cellulolyticus* ATCC 43068 **(92.08%)** – [CP000481] | Uncultured bacterium clone ncd260b10c1 **(97%)** – [HM270084.1] |
| Ap43E | 12 | KX990258.1 | *Rhodopila globiformis* DSM 161 **(92.55%)** – [D86513] | Uncultured bacterium clone Bas-7-52 **(97%) –** [GQ495410.1] |
| Ap44E | 12 | KX990259.1 | *Modestobacter versicolor* CP153-2 **(94.5%)** – [AJ871304] | Uncultured bacterium clone 3-952 **(98%)** – [KC554168] |
| Ap45E | 14 | KX990260.1 | *Conexibacter arvalis* KV-962 **(94.46%)** – [AB597950] | Uncultured actinobacterium clone UMAB-cl-13 **(98%) –** [FN811197.1] |
| Ap46E | 11 | KX990261.1 | *Frankia alni* ACN14A **(93.39%)** – [CT573213] | Uncultured bacterium clone MA03C08 **(98%)** – [FM873556.1] |
| Ap47E | 10 | KX990262.1 | *Marmoricola solisilvae* KIS18-7 **(98.84%)** – [KM199638] | Uncultured bacterium clone 11-312 **(99%) –** [KC554620.1] |

**Supp. Table 2.** Growth profile of isolates Ap25E, Ap42E, Ap38E and Ap45E on different media plates

| | Isolate Ap25E | Isolate Ap42E | Isolate Ap38E | Isolate Ap45E |
|---|---|---|---|---|
| NB 5% pH 7 | ++ | +++ | +++ | ++ |
| NB 100% pH 7 | 0 | ++ | 0 | 0 |
| TSA 5% pH 7 | 0 | +++ | 0 | 0 |
| TSA 100% pH 7 | 0 | ++ | 0 | 0 |
| R2 10% pH 7 | 0 | +++ | +++ | + |
| R2 100% pH 7 | 0 | +++ | + | 0 |
| **R2(Agar)** 100% pH 7 | 0 | 0 | 0 | 0 |
| NB 5% pH 5 | 0 | + | 0 | 0 |
| NB 100% pH 5 | 0 | 0 | 0 | 0 |
| TSA 5% pH 5 | 0 | + | 0 | 0 |
| TSA 100% pH 5 | 0 | 0 | 0 | 0 |
| R2 10% pH 5 | 0 | + | 0 | 0 |
| R2 100% pH 5 | 0 | 0 | 0 | 0 |
| **Growth temperature (NB 5%)** | | | | |
| 30°C | 0 | 0 | 0 | 0 |
| 20°C | ++ | ++ | ++ | + |
| 10°C | ++ | +++ | +++ | ++ |

+++ = good growth    ++ = moderate growth    + = weak growth    0 = no growth detected

* All plates were solidified with Gellan gum 0.7% (w/v), with exception of the R2(Agar) media, which was solidified with 1.5% (w/v) Bacto Agar
